# Effect of microplastic pollution on the gut microbiome of anecic and endogeic earthworms

**DOI:** 10.1093/femsle/fnae040

**Published:** 2024-06-07

**Authors:** Christina Papazlatani, Paolina Garbeva, Esperanza Huerta Lwanga

**Affiliations:** Department of Microbial Ecology, Netherlands Institute of Ecology (NIOO-KNAW), 6708 PB Wageningen, The Netherlands; Department of Microbial Ecology, Netherlands Institute of Ecology (NIOO-KNAW), 6708 PB Wageningen, The Netherlands; Soil Physics and Land Management Group, Wageningen University and Research, PO Box 47, 6708 PB Wageningen, The Netherlands

**Keywords:** *Aporrectodea caliginosa*, earthworms, gut microbiome, *Lumbricus terrestris*, microplastic pollution

## Abstract

Microplastic (MP) pollution constitutes an emerging type of pollution threatening both aquatic and terrestrial ecosystems. The impact on aquatic ecosystems has been extensively studied, but the effect on terrestrial ecosystems and their inhabitants is mostly underexplored. In this study, we explored the effect of MP pollution on gut bacterial microbiome of endogeic (*Aporrectodea caliginosa*) and anecic (*Lumbricus terrestris*) earthworms. The experiments were performed in sandy soil with 0.2% of low-density polyethylene MPs (LDPE MPs). We observed that the endogeic earthworms had 100% survival, while anecic earthworms survived 25 days in the control (i.e. in absence of MPs) and 21 days in the treatment with LDPE MPs. The main driver of shifts in the diversity and composition of the bacterial communities in the gut of tested earthworms was the lifestyle of the worms, followed by the presence of MPs. The bacterial microbiome diversity was significantly different among the two types of earthworms, and the highest bacterial diversity was found in the gut of the endogeic earthworms. The effect of MPs on gut bacterial microbiome was clearly observed in the changes in the relative abundance of several phyla and families of the bacterial communities in both types of earthworms, although it was most evident in the anecic earthworms. The Actinobacteriota, Proteobacteria, and Firmicutes were the main groups enhanced in the MP treatments, suggesting enrichment of the bacterial communities with potential plastic degraders.

## Teaser

Earthworm lifestyle and microplastic pollution strongly impact the structure of the earthworm’s gut microbiome.

## Introduction

Earthworms are well known as soil ecosystem engineers and soil health indicators. They are related to different nutrient cycles, nitrogen mineralization, phosphorus plant absorption, and litter decomposition. The products of their activities are generally beneficial to other soil invertebrates and to the plants, enhancing their growth and productivity (Blouin et al. 2013). Evidence also shows that earthworms contribute toward the formation of the structure of soil microbial community, either directly or indirectly (Brown and Doube 2004, Medina-Sauza et al. 2019).

Based on feeding habits, earthworms are categorized in three groups: epigeic, anecic, and endogeic. Epigeic earthworms live on the soil surface and feed from litter. Endogeic earthworms live in the soil, produce horizontal tunnels, and feed on mineral soil and decomposed material. Anecic earthworms move between soil layers making vertical burrows and feed on the litter that they drag into their burrows (Capowiez et al. 2014).

The diverse microbiome of earthworm hosts’ gut plays an important role in their nutrient metabolism, immunity, and physiology (Liu et al. 2018). Moreover, the worm casts contain bacteria from the gut that contribute to several soil ecosystem functions such as nitrogen fixation (Drake et al. 2006) or vermiremediation processes, i.e. dissipation of organic contaminants in the soil (Rodriguez-Campos et al. 2014). The food source was found to affect the earthworm gut microbiome, but the core microbiomes remained largely unchanged (Liu et al. 2018, Sapkota et al. 2020), indicating that the earthworms are key contributors of functional bacteria to the soil microbial communities, despite their lifestyle (Wang et al. 2022).

Anthropogenic soil pollution caused by the disposal of waste from industrial and urban sources or agricultural pesticides can interfere with soil microbiome and earthworm gut microbiome. An emerging type of pollution is the increasing load of microplastics (MPs; defined as plastics <5 mm) in both aquatic and terrestrial environments (EC, Scientific Opinion 6/2019). Whereas problems with MPs for functioning of aquatic ecosystems are intensively studied, their environmental impacts in the terrestrial ecosystem remain largely unexplored. A recent study revealed that both polyethylene-based and bio-based MPs can have strong effects on the assembly of the rhizosphere communities (Wesselink et al. 2023). According to the literature, agricultural land may contain more MPs than oceans. Initial quantifications suggest that background concentrations of MP might be as high as 1%–7% in agricultural and industrial soils (Rillig 2012).

The effect of MPs on earthworm behavior and on earthworm’s gut microbiome is largely unknown. Guo et al. (2023), in a literature review, discovered that most of the highly cited articles describe the toxic effects of MPs on earthworm’s growth and metabolism, including oxidative stress and DNA damage. Only a few studies focused on the shifts of the composition of earthworm’s gut microbiome after ingestions of plastic (Zhu et al. 2018, Cheng et al. 2021), usually in the presence of another pollutant like heavy metals (Wang et al. 2019, Yang et al. 2022) or polycyclic aromatic hydrocarbons (Xu et al. 2021). However, these studies were mostly performed on earthworms with an epigeic lifestyle and there is a knowledge gap on the effect of MPs on the gut microbiome of endogeic and anecic earthworms.

Based on the above, we investigated the hypothesis that the presence of MPs in soil will be reflected in the bacterial communities of the gut of both endogeic and anecic earthworms. The main aim of this study was to evaluate the effect of MP pollution (in a relatively low concentration of 0.2% low-density polyethylene) on the growth and survival of the earthworms *Aporrectodea caliginosa* (endogeic) and *Lumbricus terrestris* (anecic) and on the composition of the gut bacterial communities (gut microbiome) using 16S rRNA gene amplicon sequencing.

## Materials and methods

### Preparation of soil with MPs

Low-density polyethylene (LDPE) (Riblon, TER Hell Plastic GmbH) MPs were prepared according to Huerta Lwanga et al. (2016) with some modifications: LDPE was frozen with liquid nitrogen and fragmented manually into irregular shapes, between 100 and 150 μm, simulating the size found in soils of agricultural areas where plastic is frequently used (Huerta Lwanga et al. 2023). The MP particles were thoroughly mixed into sandy soil (26.6% brown sand, 24% silver sand, and 50% loamy silt with 0.2% organic matter) at a final concentration of 0.2% w/w (Huerta Lwanga et al. 2023) and 20% moisture content, equivalent to 3% higher than field capacity. MP concentration was chosen on account of (i) being environmentally relevant for soils under human pressure (de Souza Machaldo et al. 2019) and (ii) producing a blocking effect on water filtration in soils (Liu et al. 2022). Two hundred grams of sandy soil and sandy soil with LDPE MPs were placed in eight glass pots, respectively, totaling 16 pots (10.5 cm × 10.3 cm).

### Earthworm microcosm experiment

**Figure 1.**
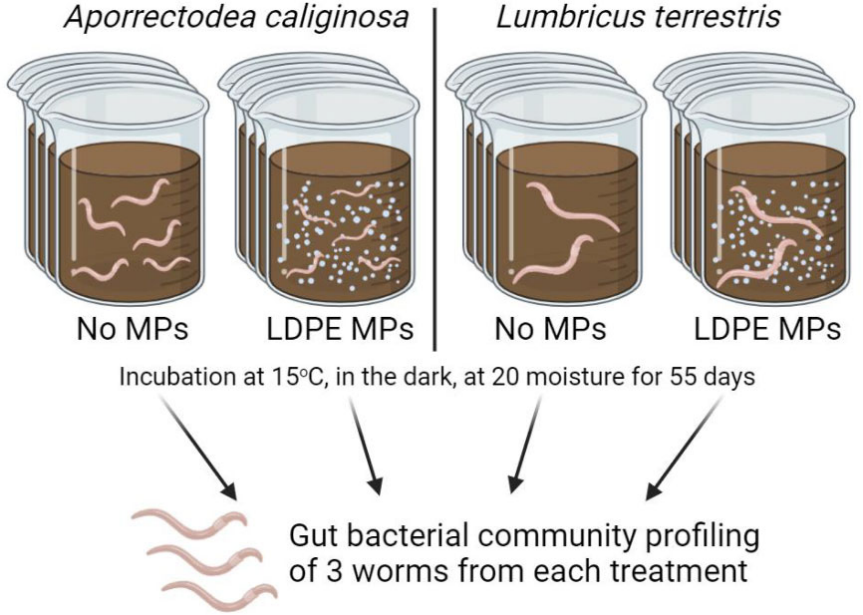
A schematic representation of the experimental setup. A total of 16 glass pots were filled with sandy soil and LDPE MPs were added into 8 of them at a concentration of 0.2% w/w. Adult earthworms belonging to *A. caliginosa* and *L. terrestris* were installed into the pots to a final number of three to five and two worms per pot, respectively. After incubating for 55 days, the gut of three worms from each treatment was collected and its bacterial community was analyzed.

*Lumbricus terrestris* and *A. caliginosa*, which correspond to anecic and endogeic lifestyles, respectively, were obtained from Wormenwekerij Wasse Company (Beilen, The Netherlands) and organic fields of Brabant, respectively. The worms were starved for 24 h before the start of the experiment, so as to have empty guts. Adult earthworms were installed separately per glass pot to a total initial biomass per pot of 8.18 ± 1.2 g for *L. terrestris* and 1.75 ± 0.07 g for *A. calaginosa*, which corresponded to a final number of two anecic worms per pot and three to five endogeic worms per pot. (Fig. 1). Then the pots were incubated at 15°C, in darkness, since the earthworms are photosensitive. The soil moisture content was maintained at 20 ± 2% (average environmental conditions observed in the Netherlands during autumn) by weighting the pots at the beginning of the trial and by the use of a time-domain reflectometry (TDR) moisture sensor. Earthworm incubation time was 55 days, in order to assure intestinal bacteria adaptation to the new conditions (minimum 49 days; Chao et al. 2021).

### Earthworm measurements

The weight of the earthworms was recorded before placement in the glass pots and at the end of the incubation period, for the earthworms that were still alive. Once the weight was recorded, the earthworms were frozen at −18°C for assessment of the gut microbiome. If the earthworms were found dead, they were immediately frozen and their weight was not recorded for avoiding contamination, since their body was very fragile. The gain and loss of weight of the earthworms that were alive till the end of the incubation period was calculated according to Equation (1), where *K*_gr_ is the growth rate, *Morg*_1_ is the initial individual weight, *Morg*_2_ is the individual weight at the end of the incubation period, and *t* is the experimental time.


(1) \begin{eqnarray*}
K\mathrm{ _{ gr}} = \frac{{\frac{{\textit{Morg}2\, -\, \textit{Morg}1}}{{\textit{Morg}1}}}}{t}.
\end{eqnarray*}

### Statistical analysis

Significant differences of the earthworms’ growth rate among treatments were evaluated with the *t*-test, once the normality of the data was verified, or the nonparametric Mann–Whitney U test (or Wilcoxon rank-sum test) if the data did not follow the normal distribution.

### Earthworm’s gut microbiome DNA extraction and sequencing

In sterile conditions (UV cabinet), earthworms were opened according to Huerta Lwanga et al. (2018) for extracting the gut content, which was immediately frozen at −18°C. DNA was extracted from the earthworm gut samples using the DNeasy PowerSoil Pro Kit (Qiagen) following the manufacturer’s instructions. Extracted DNA quality was assessed via electrophoresis on 0.8% agarose gel and DNA concentration was determined with a NanoDrop spectrophotometer (Thermo Fisher Scientific). Subsequently, DNA was subjected to amplicon sequencing of the V3–V4 hypervariable regions of the 16S rRNA gene using Illlumina MiSeq 300 paired-end technology in the sequencing center BaseClear B.V., Leiden, Netherlands. The amplification was performed with the primer set 341f 5′-CCTACGGGNGGCWGCAG-3′ and 785r 5′-GACTACHVGGGTATCTAATCC-3′ (Thijs et al. 2017).

### Earthworm’s gut microbiome analysis

Primers were removed from the amplicon sequences with the “Cutadapt” tool (version 4.3; Martin 2011) and the “dada2” package (version 1.26.0; Callahan et al. 2016) of the R language for statistical computing (version 4.0.5; released 2021–03-31) (R Core Team 2020), following the instructions in DADA2 ITS Pipeline Workflow (version 1.8). Initial quality control showed that read 2 contained several low-quality base pairs, which, when trimmed, led to a reduced number of high-quality reads and merged sequences compared to running the analysis with only read 1. Therefore, bacterial community analysis was performed with high-quality read 1 sequences. Read 1 sequence data are publicly available in the National Centre for Biotechnology Information (NCBI) under BioProject accession number PRJNA1051054.

After primer removal, the reduced amplicon sequences were subjected to quality screening, chimera removal, generation of the amplicon sequence variant (ASV) abundance matrices, and taxonomic classification with the dada2 package. Silva prokaryotic small subunit (SSU) rRNA gene taxonomic training data formatted for “dada2”, version 138.1, were used for the classification of the amplicon sequences. ASVs that were classified as mitochondria or chloroplasts and ASVs that were present in only one sample were removed from further analysis. Microbial diversity coverage was assessed via rarefaction curves prepared with the R package “vegan” (version 2.6.4; Oksanen et al. 2019) and the percentage of total species represented in a sample (Good 1953) was estimated with the R package “entropart” (version 1.6.13; Marcon and Hérault 2015). The bacterial communities’ α-diversity was evaluated by calculating the observed richness (S), Pielou’s evenness index (Pielou 1966), Shannon index (Spellerberg and Fedor 2003), and Gini–Simpson index (Roswell et al. 2021) with the R package “microbiome” (version 1.21.1; Lahti and Shetty 2012). The difference of the α-diversity indices between presence and absence of MPs for each earthworm was assessed with pairwise comparison using the *t*-test, if the indices in test were normally distributed, or the Wilcoxon rank-sum test if not. Canonical correspondence analysis (CCA; ter Braak and Verdonschot 1995) or redundancy analysis (Israels 1984), accompanied by permutational analysis of variance, was used to estimate the β-diversity between treatments for each earthworm. The analysis to be performed was determined by the first axis length of detrended correspondence analysis (Lepš and Šmilauer 2003), which was carried out on Hellinger-transformed abundance matrices (Legendre and Gallagher 2001). Aforementioned analyses were conducted with the R package “vegan”. Lastly, differential abundance analysis was performed with the nonparametric Kruskal–Wallis test after transforming the ASV abundances with robust centered log ratio (rclr) (Martino et al. 2019) of the “decostand” command of the “vegan” R package.

## Results and discussion

### Earthworm survival and growth rate

All anecic earthworms, regardless of MP treatment, were found dead at the end of the incubation period (day 55); therefore, growth rates were calculated only for the endogeic earthworms. In our experiment, the anecic earthworms lived for 25 days in the control and for 21 days in the treatment with LDPE MPs. The anecic earthworms normally feed on soil and litter, and in order to provide similar conditions for both earthworm categories, both categories did not have litter on the soil surface. Feeding conditions are well known to influence earthworm’s performance (Liu et al. 2018).

**Figure 2.**
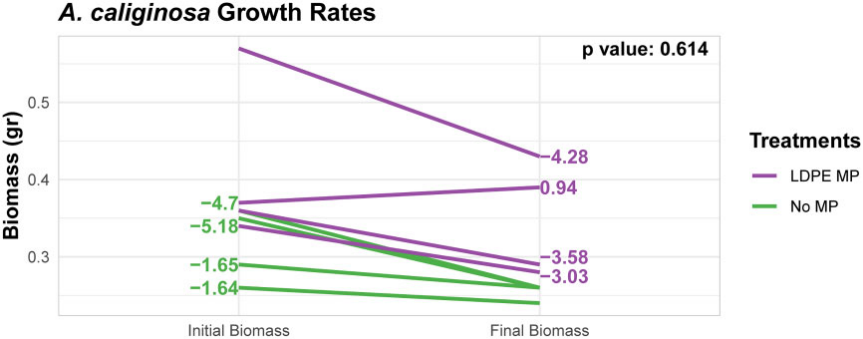
Growth rate of endogeic earthworms in the absence and presence of LDPE MPs. Growth rates for each earthworm are presented for treatments without LDPE MPs (left) and for treatments with LDPE MPs (right). On the top right, the *P*-value of pairwise comparison test of the growth rates between the two MP treatments is presented.

All in all, endogeic earthworms lost weight after 55 days, which is evident by the negative growth rates (Fig. 2), and LDPE MP treatment did not seem to have an effect on the weight loss of endogeic earthworms (*P*-value .614) (Fig. 2). The lack of effect from the LDPE MPs could be due to the low concentration of the MPs used in the present research. Several studies have also shown that the adverse effect of MPs on earthworm growth and behavior is only present in higher concentrations of MPs. Cao et al. (2017) demonstrated that the growth and viability of the epigeic earthworm, *Eisenia fetida*, was not affected in the presence of low concentrations of polystyrene MPs (0.25% and 0.5% w/w). Similarly, Ding et al. (2021) revealed that the presence of low concentrations of biodegradable (polylactic acid and polypropylene carbonate), and the nonbiodegradable (polyethylene) MPs (<125 g/kg) did not have an effect on the growth and survivability of *E. fetida*.

In this study, moisture conditions were similar to those occurring on the 0–20 cm top soil layer of west Netherlands (van der Velde et al. 2023) and lower than those reported by Wesselink et al. (2023) for 10–15 cm top soils from organic agricultural field tillage (28%) and nontillage (27%) systems. Those environmental factors, even though realistic, might have been responsible for the weight decrease of endogeic earthworms. Wever et al. (2001) demonstrated that the survival and growth of the endogeic species *Aporrectodea tuberculate* was negatively influenced by the reduction in soil moisture (25%, 20%, 15%, and 10% moisture content), in all tested temperatures (5°C, 10°C, 15°C, and 20°C). Likewise, Eriksen-Hammel and Whalen (2006) also showed that lower soil water content, resulted in lower growth rates for the endogeic species *A. caliginosa*.

### Bacterial diversity in earthworm gut

The sequencing effort of the earthworms’ gut microbiome yielded 19 956–28 874 amplicon sequences, which resulted in 11 849–20 476 high-quality sequences after quality control analysis (Supplementary Table 1). The sample sizes correspond to the plateau of the rarefaction curves (Supplementary Fig. 1), and the Good’s coverage index for all samples is 1 (Supplementary Table 1), indicating adequate coverage of the bacterial species richness.

**Figure 3.**
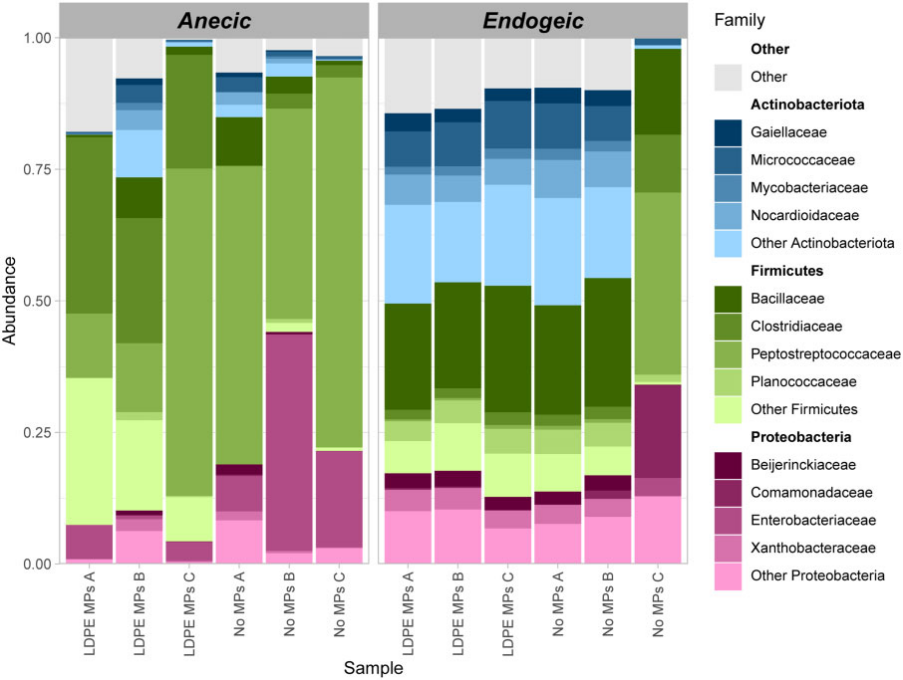
The gut bacterial community composition at the family level of anecic (*L. terrestris*) and endogeic (*A. caliginosa*) earthworms when grown in the presence (LDPE MPs) and absence (No MPs) of MPs.

The gut microbiome composition of anecic and endogeic earthworms was diverse, and variation was observed between the replicates of each earthworm. The bacterial communities of the *L. terrestris* gut were dominated by Firmicutes (70% ± 15.06), and more specifically by *Peptostreptococcaceae, Clostridiaceae*, and *Enterobacteriaceae* families, which, on average, accounted for 42.4% ± 25.1%, 14.1% ± 14.0%, and 7.1% ± 11.1% of the total microbial community, respectively (Fig. 3, Supplementary Table 2). Other highly abundant groups were *Enterobacteriaceae* and *Micrococcaceae* families, which accounted for 12.8% ± 15.1% and 1.4% ± 1.4%, respectively. The gut bacterial communities of *A. caliginosa* comprised mostly of Firmicutes, Actinobacteriota, and Proteobacteria (also known as Pseudomonadota), with average relative abundances in both treatments, of 40.8% ± 11.6%, 31.0% ± 14.4%, and 18.6% ± 7.8%, respectively (Fig. 3, Supplementary Table 2). Contrasting to anecic earthworms, *Bacillaceae* and *Micrococcaceae* families were highly abundant with 21% ± 3% and 6.8% ± 2.8% average relative abundances in both treatments (Fig. 3, Supplementary Table 2). The observed dominance of Proteobacteria, Firmicutes, and Actinobacteriota in both anecic and endogeic earthworms is in agreement with other studies. For example, Sapkota et al. (2020) showed that the bacterial communities of the gut of earthworms with different lifestyles (endogeic and anecic) were dominated by Proteobacteria, Actinobacteria, and Firmicutes, whereas Liu et al. (2018) observed that only Proteobacteria were the dominant taxa of the gut microbial communities of *E. fetida*, an epigeic earthworm.

### α-diversity of earthworms gut bacterial communities

**Figure 4.**
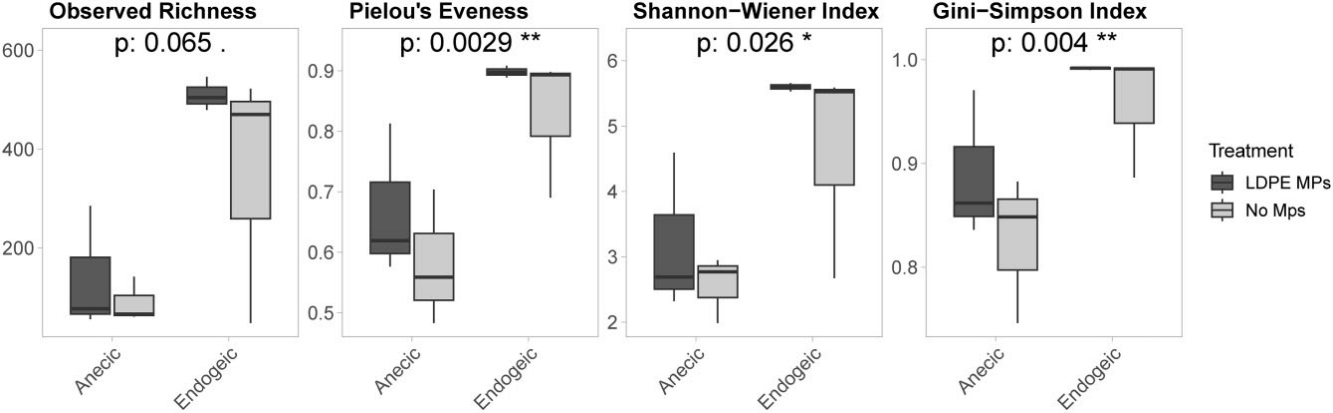
The α-diversity indices of anecics (*L. terrestris*—left) and endogeic (*A. caliginosa*—right) earthworms gut microbiome in the presence and absence of MPs. *P*-values at the top of each panel indicate the significance of pairwise comparison between the earthworms of the α-diversity indices of both treatments.

*Aporrectodea caliginosa* hosted microbial communities with an average number of ASVs (observed richness) of 428.2 ± 188.3 in both treatments. The bacterial composition was even and diverse, with Pielou’s evenness index at 0.86 ± 0.08 and Shannon’s and Gini–Simpson’s diversity indices at 5.10 ± 1.19 and 0.97 ± 0.04, respectively (Fig. 4). On the other hand, *L. terrestris* hosted a significantly less rich and diverse gut microbiome compared to the endogeic earthworm *A. caliginosa*. The observed number of ASVs was limited to 114.5 ± 89.3 species in both MP treatments and Pielou’s evenness was estimated at 0.63 ± 0.12 indicating that the members of the gut microbial communities do not share similar abundances. Shannon’s and Gini–Simpson’s diversity indices were 2.88 ± 0.91 and 0.86 ± 0.07, respectively (Fig. 4). Pairwise comparison showed that the α-diversity indices of the gut microbiome were notably different between the two earthworms (*P-*value < .05, Fig. 4), although, no effect was evident of the MPs treatment in each earthworm (*P*-value > .05, data not shown). The lack of effect of the presence of MPs could be attributed to the low amount of MPs in the soil. Similar results were obtained by Zhu et al. (2018) who tested the influence of various concentrations of polystyrene MPs (0%, 0.025%, 0.5%, and 10% polystyrene–oatmeal mixture) on the gut microbiome composition of the epigeic earthworm *Enchytraeus crypticus*. The diversity of the microbiome was not affected by the presence of polystyrene MPs at low concentrations, whereas it declined significantly at the highest tested concentration (10%) (Zhu et al. 2018). In the same manner, Cheng et al. (2021) demonstrated that the presence of 0.25% high-density polyethylene (HDPE) or polypropylene (PP) MPs did not affect significantly the richness and diversity of the bacterial communities of the anecic earthworm *Metaphire guillelmi* compared to the earthworms grown without MPs.

### Causes of dissimilarity of earthworms’ gut microbiome

**Figure 5.**
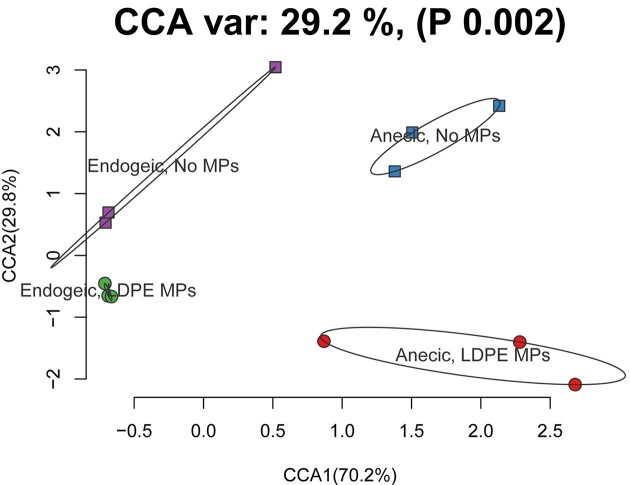
CCA of the gut bacterial communities of the anecic earthworm *L. terrestris* and the endogeic earthworm *A. caliginosa* when exposed to MPs (circle, ○) or not (square, □).

CCA revealed that both species and MP treatments accounted for 29.2% of the total variation of the gut microbiomes (Fig. 5). On the first axis, which accounted for 70.2% of variation, it is evident that the samples are separated according to earthworm species, whereas on the second axis, which accounts for 29.8% of variation, the samples are separated according to MPs treatment (Fig. 5). Hence, the anecic and endogeic life form cause stronger dissimilarity in the gut microbiome. Our results are consistent with the study of Sapkota et al. (2020), in which they found that the earthworm life-form predominately shapes the gut bacterial community structure.

**Figure 6.**
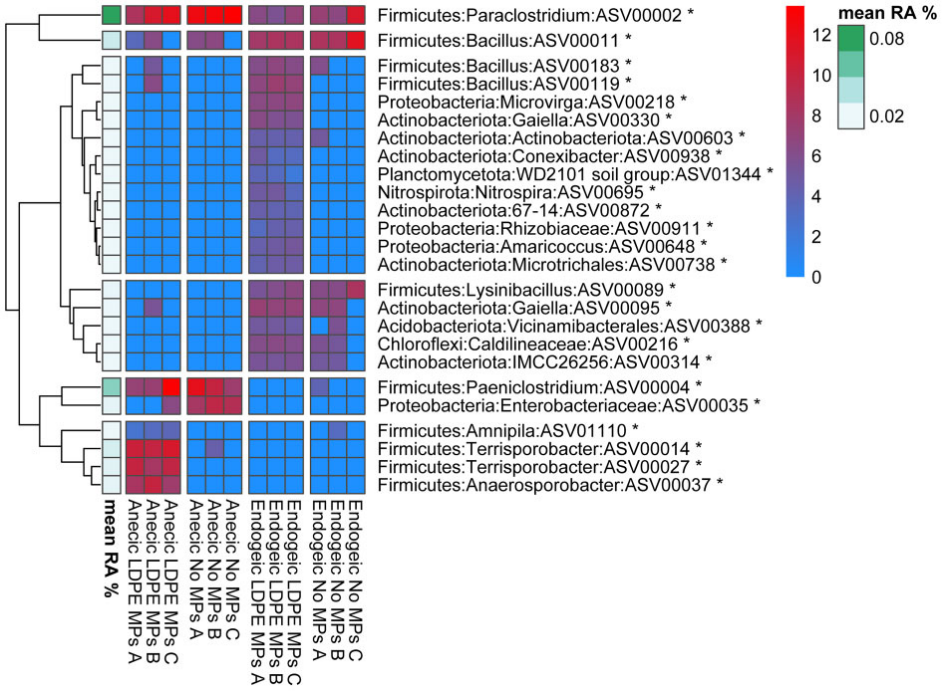
Heatmaps of the log-transformed abundances of 25 bacterial ASVs in the gut of the anecic earthworm *L. terrestris* and the endogeic earthworm *A. caliginosa* whose abundance was significantly different between each tested earthworm and MP treatment. ASVs are clustered according to Euclidean distances. The stars in the ASVs’ annotation denote the level of significance (*** for *P* < .001, ** for *P* < .01, and * for *P* < .05).

The shift of the ASVs’ relative abundance between the different microbiomes, was further explored on account of their distinct separation in the CCA analysis. Differential abundance analysis of the rclr-transformed abundances between each tested earthworm and MP treatment was performed with the nonparametric Kruskal–Wallis test. *Clostridia* were found at significantly higher relative abundance in *L. terrestris*, such as *Paraclostridium bifermentans* (ASV 00002), *Terrisporobacter glycolicus* (ASV 00014 and 00027), *Paeniclostridium* sp. (ASV 00004), *Anaerosporobacter* (ASV 00037), *Amnipila* (ASV 01110), and *Clostridium sensu stricto* 1 (ASV 00015) and 13 (ASV 00012, 00026, and 00040), as well as the ASV 00035, a member of the *Enterobacteriaceae* family (Fig. 6, Supplementary Fig. 2). The vast majority of the ASVs that were found in significantly higher relative abundance in *L. terrestris* (excluding ASV 00035) belonged to the Firmicutes phylum. Our findings are in agreement with the ones from Sapkota et al. (2020), who compared the OTUs of the gut bacterial communities between the anecic earthworm *Lumbricus herculeus* and the endogeic earthworms *Allolobophora chlorotica, A. caliginosa*, and *Aporrectodea tuberculata* and they observed that the majority of the OTUs that were in significantly higher abundances in *L. herculeus* are Firmicutes. Out of all the bacteria that were found in significantly higher relative abundance in *L. terrestris*’ gut, ASV0002 and ASV00035 were found to be in substantially higher relative abundance in the absence of LDPE MPs, whereas the relative abundance of ASVs 00012, 00014, 00015, 00026, 00027, 00037, and 00040 was increased in the presence of LDPE MPs (Fig. 6, Supplementary Fig. 2). Unlike our findings, Cheng et al. (2021) who explored the effect of HDPE and PP MPs on the diversity and community structure of the gut microbiota of the anecic earthworm, *M. guillelmi*, reported stable gut microbial communities that were not affected by the presence of MPs. Similarly, Wang et al. (2019) upon examination of the gut bacterial communities of the anecic earthworm *Metaphire californica* when exposed to polyvinyl chloride MPs, observed no significant differences comparing to untreated control. On the other hand, Zhu et al. (2018) in their study of the effect of different polystyrene MP concentrations on the gut microbiome of the epigeic earthworm *E. crypticus*, observed that the presence of 10% MPs resulted in increase in the relative abundance of members of the Bacteroidetes phylum and a decrease in members of the Proteobacteria and Planctomycetes phyla. Several ASVs were present only in the endogeic species, *A. caliginosa*, with or without MP treatments such as seven ASVs of the *Actinobacteriota* phylum (ASV 00314, 00426, 00521, 00583, 00602, 00603, and 00754), three ASVs that belong to *Chroflexi* orders KD4-96 and *Anaerolinae* (ASV 00139, 00409, and 00216), five ASVs classified in *Bacillales* (ASV 00089, 00154, 00158, 00166, and 00709), two ASVs in *Paenibacilalles* (ASV 00091 and 00132), five *Alphaproteobacteria* ASVs of the *Rhizobiales* order (ASV 00080, 00100, 00178, 00249, and 00292), and the ASVs 00266 (*Verrucomicrobiae*/*Chthoniobacter* sp.), 00388 (*Acidobacteriota*/*Vicinamibacterales*), 00565 (*Planctomycetes*/*Pir4* lineage sp.), and 00711 (*Burkholderia*–*Caballeronia*–*Paraburkholderia* sp.). The composition of the bacterial communities of the endogeic earthworms have been less studied comparing to epigeic and anecic worms. Nevertheless, similar to our findings, Thakuria et al. (2010) in their study of bacteria that inhabit the walls of the gut of both endogeic and anecic earthworms, found that the endogeic earthworms *A. callaginosa* and *A. longa* contained species that belonged in the Proteobacteria and Actinobacteriota phyla.

Lastly, quite a few ASVs were detected in the gut microbiome of *A. caliginosa*, only in the presence of LDPE MPs, such as four ASVs belonging to the *Actinobacteriota* phylum (ASV00330—*Gaiella* sp, ASV00738—*Microtrichalles*, ASV00872—*Solirubrobacterales* 67–14 family, and ASV00938—*Conexibacter* sp.), three *Alphaproteobacteria* (ASV00218—*Microvirga* sp., ASV00911—*Rhizobiaceae* family, and ASV00648—*Amaricoccus* sp.), ASV01344 (Planctomycetota/*WD2101* soil group), and ASV00695 (*Nitrospira defluvii*).

All in all, the ASVs that were found in higher relative abundance in the presence of LDPE MPs belonged to the Firmicutes phylum for *L. terrestris*, and Proteobacteria, Plactomycetota, Actinobacteriota, and Nitrospirota for *A. calaginosa*. The shift of the bacterial communities that were induced by the MPs could lead to enrichment of communities with bacterial families that participate in the depolymerization of MPs. Arpia et al. (2021) in their literature review on MP degradation, displayed a set of bacteria that are able to degrade MPs, most of which belonged to the Firmicutes, Proteobacteria, and Actinobacteria phyla, and more specifically to the *Bacillaceae, Enterobacteriaceae, Nocardiaceae*, and *Streptomycetaceae* families. Moreover, in a previous study, Huerta Lwanga et al. (2018) isolated from the gut of *L. terrestris* six bacterial strains that when applied to LDPE MP contaminated soil, were able to reduce the concentration and the size of MPs. Two of the plastic-deteriorating bacteria were classified as Firmicutes (*Bacilaceae*) and the remaining four as Actinobacteria (*Microbacteriaceae, Nocardiaceae, Mycobacteriaceae*, and *Streptomycetaceae*) (Huerta-Lwanga et al. 2018).

In this study, we demonstrated that both the lifestyle of the worms and the presence of MPs, were responsible for shifts in the diversity and composition of the bacterial communities in the gut of anecic and endogeic earthworms, with lifestyle being a stronger influence. Previous studies on the effect of MPs on earthworms’ gut microbiome were contradictory, with some of them reporting strong influence, whereas others no significant effects. The great diversity of research findings, could have resulted from the diverse parameters that were studied, including the earthworms’ species and lifestyles, the material of the plastics, their physicochemical properties (shape, size, and additives), and the exposure concentration, as well as the incubation conditions (soil moisture content and temperature). Therefore, more research is needed in order to understand the mechanisms and effects of MP pollution in the gut microbiome, and overall performance of earthworms in MP contaminated soil.

Overall, the incubation conditions used in this study resulted in lack of viability for *L. terrestris*, and loss of weight for *A. calaginosa*. The presence of LDPE MPs in the soil did not seem to have an effect on the growth and survival of the earthworms, which could be due to the low concentration of MPs applied in the soil (0.2% w/w). Furthermore, by using DNA amplicon sequencing we have demonstrated that the richness and diversity of the gut microbiomes of earthworms are strongly affected by its host life form, while MP presence contributed to the variation at a smaller extent. Higher bacterial diversity was detected in the endogeic earthworm, *A. calaginosa*, whose bacterial community was dominated by Firmicutes, Actinobacteriota, and Proteobacteria. On the contrary, the bacterial community of the *L. terrestris* gut was dominated by Firmicutes. The differences in gut microbiomes of anecic and endogenic earthworms are considerably linked to their lifestyle, performance, and survival rates. Our results also revealed clear shifts of the gut microbiome in response to MP treatment with Actinobacteriota, Proteobacteria, and Firmicutes as the main groups enhanced by the MP treatments, which could result to enrichment of the bacterial communities with potential plastic degraders.

## Supplementary Material

fnae040_Supplemental_Files
