# Supplementary material for: Effect of microplastic pollution on the gut microbiome of anecic and endogeic earthworms
Source: FEMS Microbiol Lett. 2024 Jun 7;371:fnae040. doi: 10.1093/femsle/fnae040 (PMC11232513; doi:10.1093/femsle/fnae040)
Supplement: fnae040_Supplemental_Files [file fnae040_supplemental_files.zip › Supplementary Figures.docx]

**Supplementary Figures**


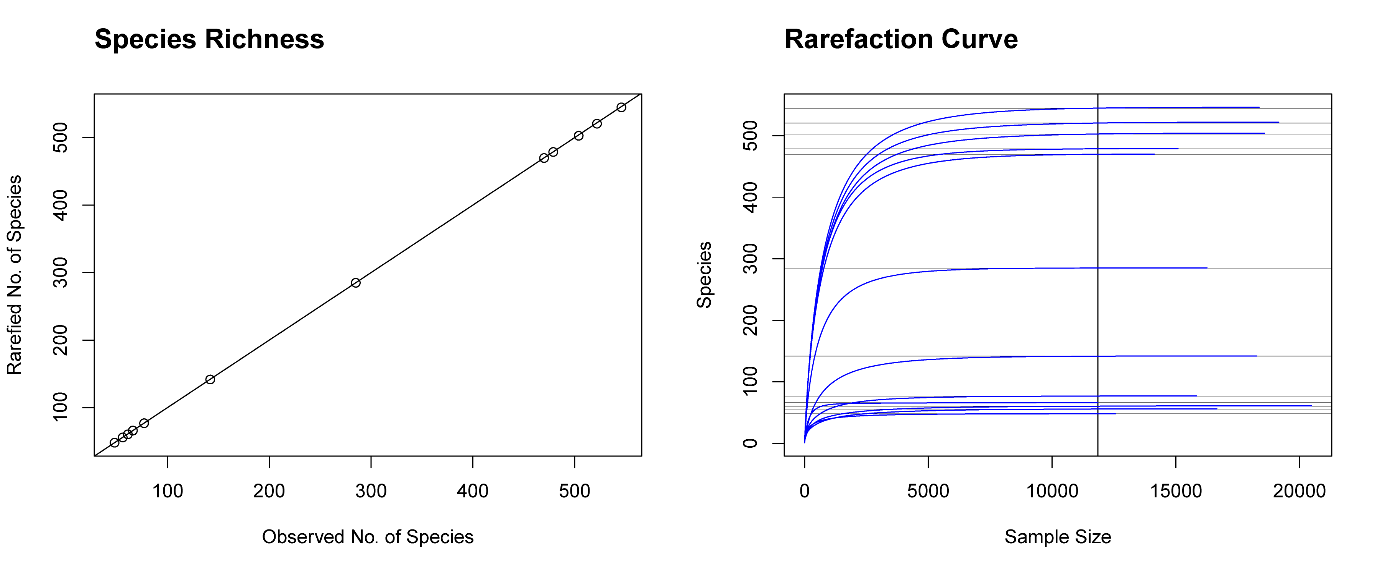


**Supplementary Figure 1.** Rarefied and observed number of species richness (left) and samples’ rarefaction curves (expected species richness in random subsamples of each sample size) (right)
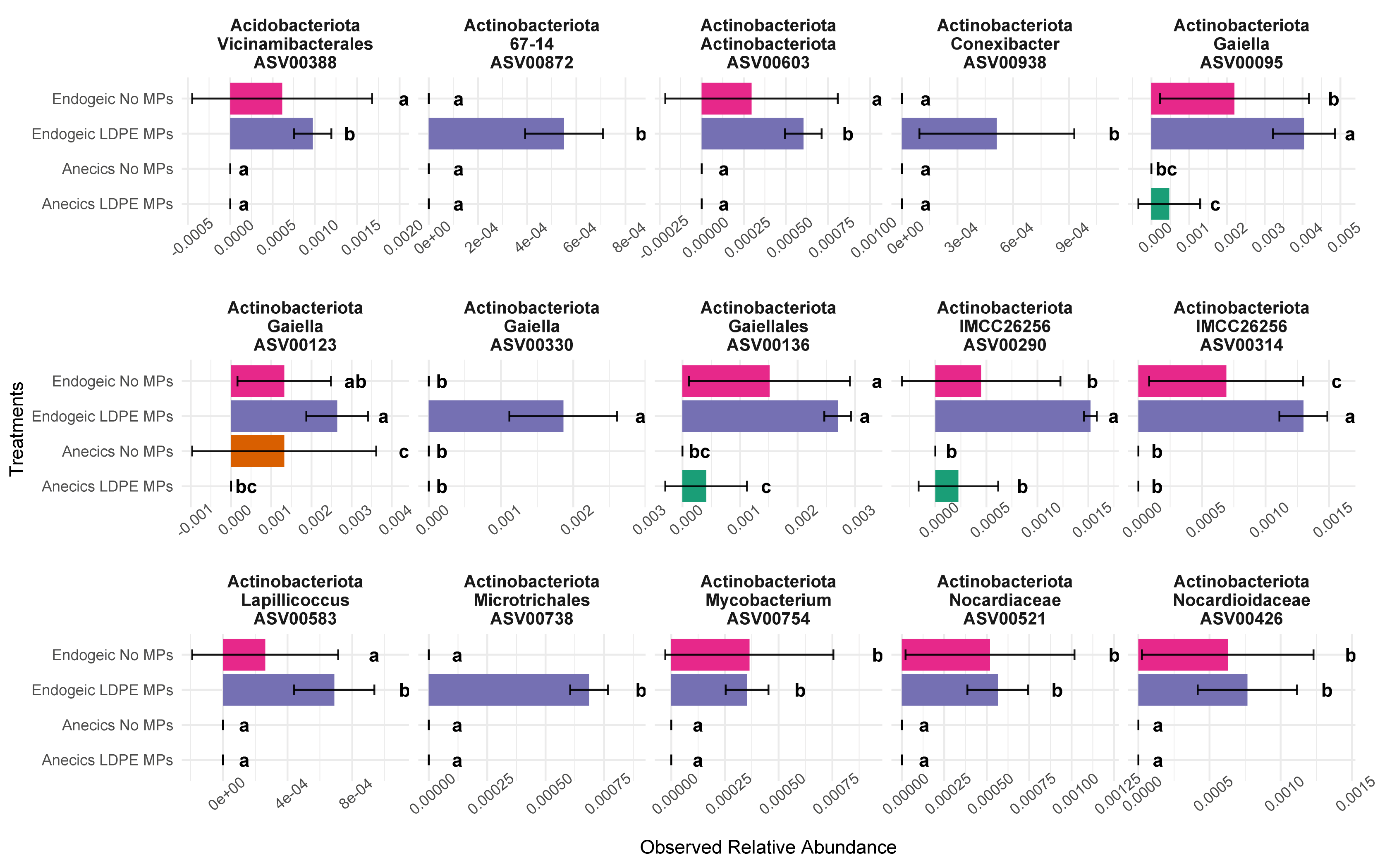

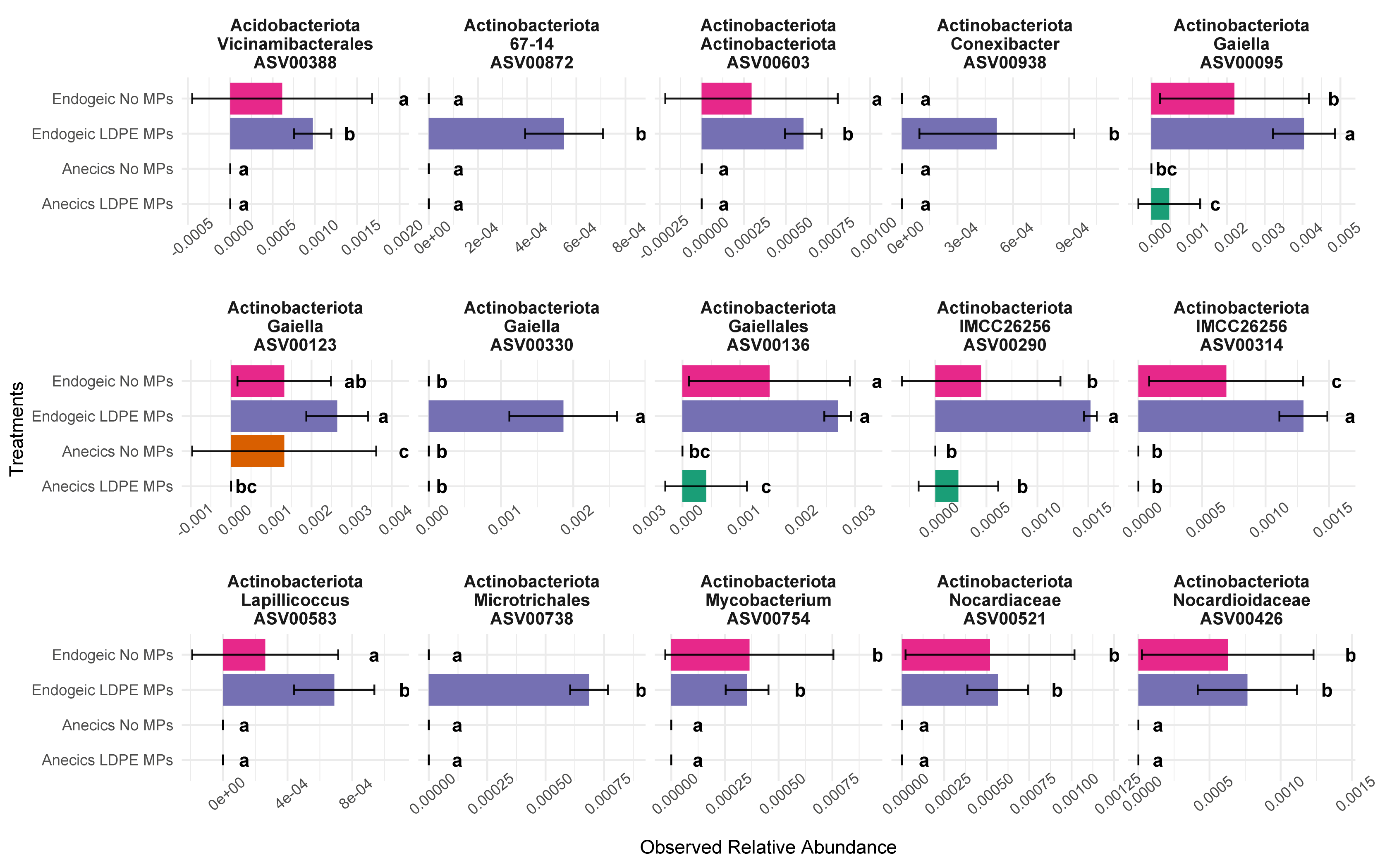

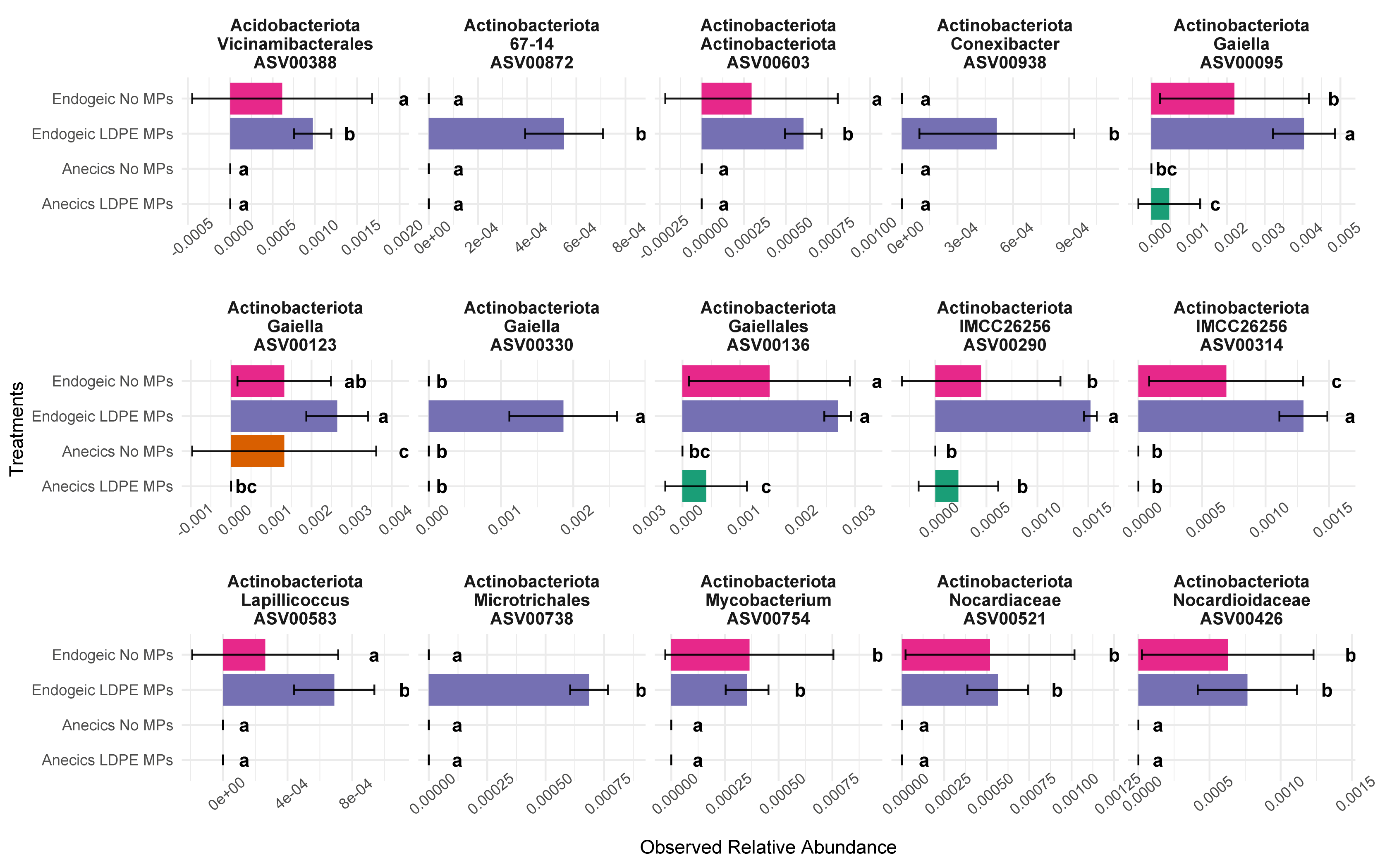

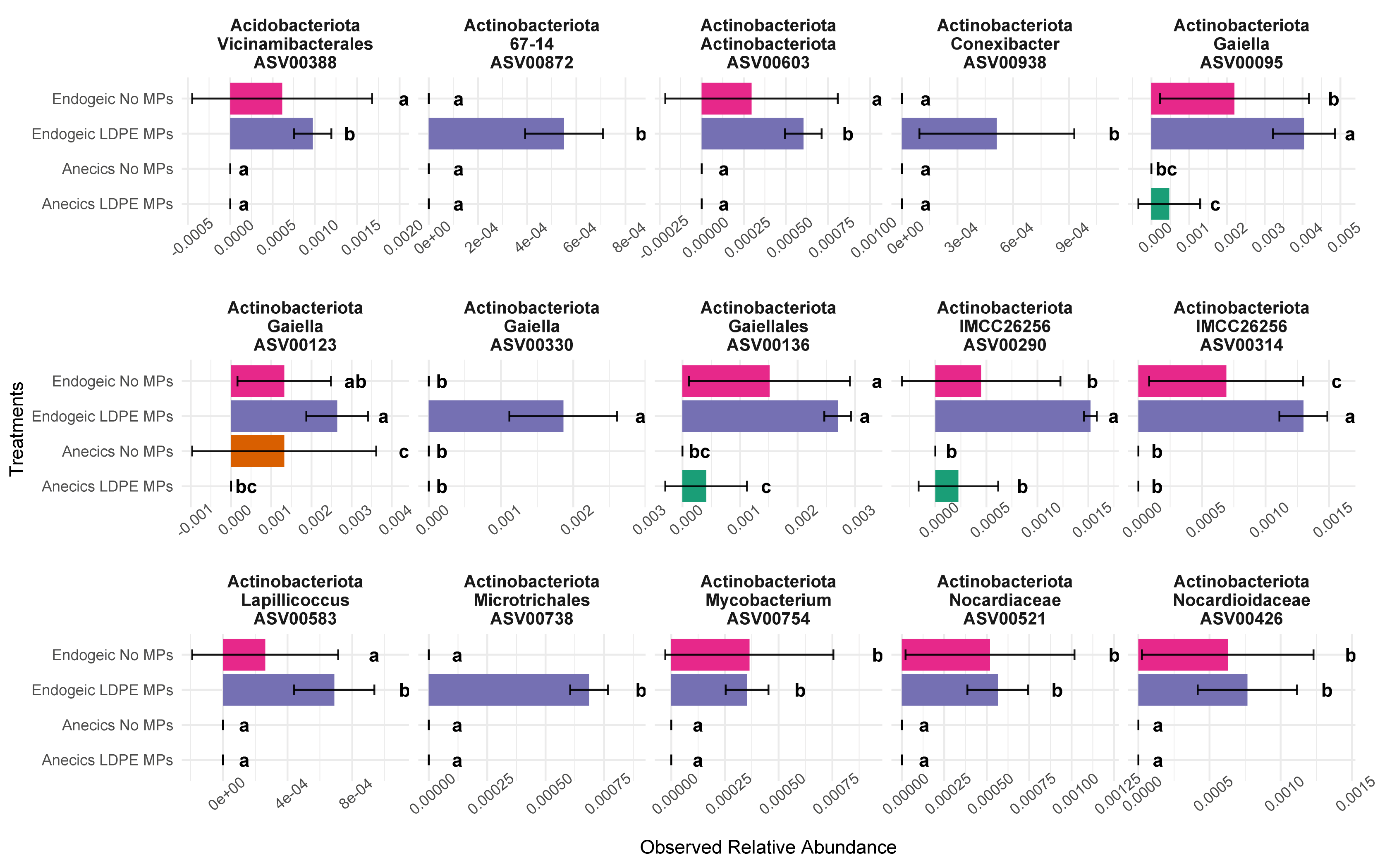

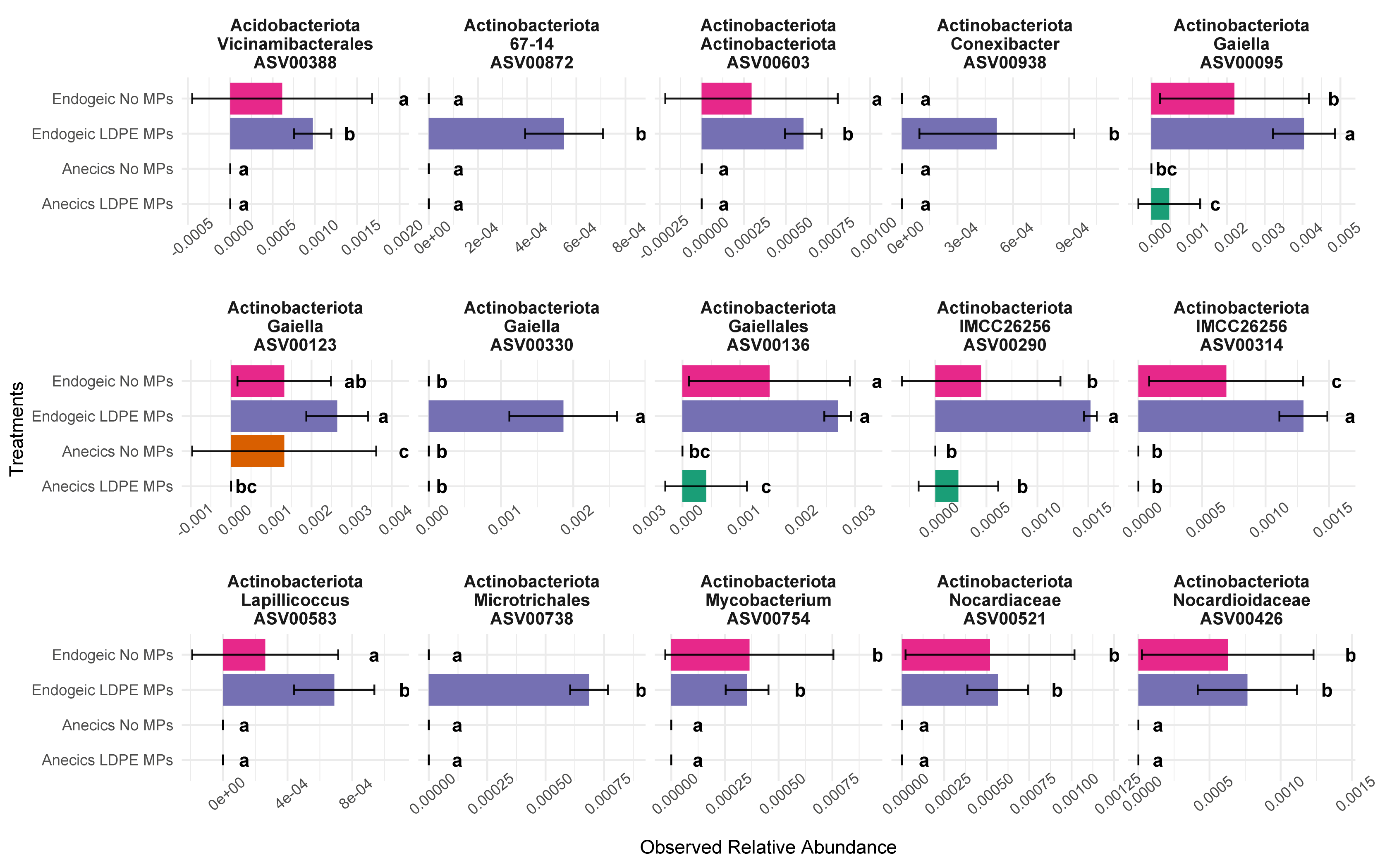


**Supplementary Figures 2.** Barplots of the observed relative abundance of bacterial ASVs in the gut of the anecic earthworm *L. terrestris* and the endogeic earthworm *A. calaginosa* whose abundance was significantly different between each tested earthworm and MP treatment.
