# Supplementary material for: Effect of microplastic pollution on the gut microbiome of anecic and endogeic earthworms
Source: FEMS Microbiol Lett. 2024 Jun 7;371:fnae040. doi: 10.1093/femsle/fnae040 (PMC11232513; doi:10.1093/femsle/fnae040)
Supplement: fnae040_Supplemental_Files [file fnae040_supplemental_files.zip › Supplementary Table 1 - Sequence Numbers revised final.docx]

| **Earthworm** | **Treatments** | **Replicate** | **Input** | **High Quality Sequences** | **Coverage** |
| --- | --- | --- | --- | --- | --- |
| Endogeic  *(A. caliginosa)* | LDPE MPs | A | 22842 | 15088 | 1 |
|  |  | B | 27709 | 18361 | 1 |
|  |  | C | 28090 | 18582 | 1 |
|  | No MPs | A | 28874 | 19156 | 1 |
|  |  | B | 22214 | 14130 | 1 |
|  |  | C | 25543 | 12557 | 1 |
| Anecics  *(L. terrestris)* | LDPE MPs | A | 26358 | 16655 | 1 |
|  |  | B | 27192 | 16259 | 1 |
|  |  | C | 26989 | 15838 | 1 |
|  | No MPs | A | 19956 | 11849 | 1 |
|  |  | B | 24282 | 18266 | 1 |
|  |  | C | 25911 | 20476 | 1 |

**Supplementary Table 2.** Number of raw amplicon sequences (Input) and of high-quality sequences after quality control analysis
